# Prevalence and virulence potential of *Aeromonas* spp. isolated from human diarrheal samples in North East Italy

**DOI:** 10.1128/spectrum.00807-23

**Published:** 2023-10-19

**Authors:** Giulia Bernabè, Paola Brun, Giuseppe Di Pietra, Veronica Zatta, Shirin Asad, Silvia Meneghello, Giampaolo Cordioli, Enrico Lavezzo, Elisabetta Valente, Sofia Mietto, Valeria Besutti, Ignazio Castagliuolo

**Affiliations:** 1 Department of Molecular Medicine, University of Padova, Padova, Italy; 2 Microbiology Unit of Padua University Hospital, Padova, Italy; University of Nebraska-Lincoln, Lincoln, Nebraska, USA

**Keywords:** toxins, intestinal epithelial barrier, enteropathogens, antibiotic resistance, biofilms, emerging pathogens, adhesion

## Abstract

*Aeromonas* spp. are emerging human pathogens causing intestinal and extra-intestinal infections. Since their relevance in Western Europe as gastrointestinal pathogens is not well established, we investigated *Aeromonas* spp. prevalence in diarrheal fecal samples in an Italian University Hospital and characterized the virulence mechanisms of the isolates. *Aeromonas* spp. isolated from diarrheic stools using standard culture methods were identified by molecular techniques. Antimicrobial resistance was assessed by the micro broth dilution. Toxins, flagella, and type III secretion system genes were evaluated by polymerase chain reaction. Biofilm was quantified by crystal-violet staining. Interaction with human intestinal epithelial cells (Caco-2) was assessed by quantifying adhesion, interleukin (IL)-8 secretion, and epithelial barrier integrity. *Aeromonas* spp. represented 20.6% of bacterial pathogens isolated from diarrheic feces, the second most common enteropathogen. *A. cavieae* constituted 75% of the identified species, showing a relatively low clustering value. About 52% of *Aeromonas* isolates showed resistance to amikacin, whereas only 7.5% showed multiple drug resistance. Four or more virulence genes were identified in 66.7% of *A. cavieae* isolates and 100% of *A. dakensis. Aeromonas* isolates (82.5%) showed moderate or important biofilm-producing ability. Adhesion to Caco-2 cells correlated to *fla*+ gene, whereas *ascV+* and *aexU*+ strains significantly induced IL-8 release from Caco-2. *Aeromonas aer+* strains caused ZO1 and occluding redistribution and a significative reduction in trans-epithelial resistance. *Aeromonas* spp. emerge as relevant human intestinal pathogens with a disparate arsenal of pathogenicity factors causing diarrhea through different mechanisms.

**IMPORTANCE:**

In this work, we demonstrate the epidemiologic relevance of the *Aeromonas* genus as the cause of infective diarrhea in North East Italy, both in children and adult subjects, with the significative presence of highly pathogenic strains. *Aeromonas* strains possess a heterogeneous armamentarium of pathogenicity factors that allows the microbe to affect a wide range of human intestinal epithelial cell processes that justify the ability to induce diarrhea through different mechanisms and cause diseases of variable severity, as observed for other gastrointestinal pathogens. However, it remains to be determined whether specific genotype(s) are associated with clinical pictures of different severity to implement the diagnostic and therapeutic approaches for this relevant enteric pathogen.

## INTRODUCTION

*Aeromonas* spp. are widespread bacteria inhabiting a variety of environmental niches, including soil and aquatic environments and colonizing aquatic and terrestrial animals (1). Consequently, it is not surprising that microbes of the genus *Aeromonas* have been isolated from drinking water supplies and food items such as meat, milk, dairy products, and vegetables (2, 3). Granting that Aeromonads are well-recognized disease-causing pathogens in fish and other cold-blooded organisms, these microbes have emerged as relevant human pathogens in the past few years (4). Clinical and experimental studies have demonstrated that Aeromonads, coming into the human body through contact or ingestion of polluted water or food, cause various infections in both immunocompetent and immunocompromised subjects (4, 5). *Aeromonas* spp. have been implicated in extra-intestinal diseases, such as septicemia, soft-tissue wounds, eyes, and respiratory tract infections. However, the most common human infections involve the gastrointestinal tract (5, 6). Consistent with the ability of Aeromonads to colonize water supply systems and foods, gastrointestinal diseases caused by these pathogens are relevant in developing countries. Nevertheless, *Aeromonas* spp. infections in industrialized countries have been reported too, but the real relevance is not well documented (7, 8).

Numerous *Aeromonas* species can be identified from the environment and foods. However, only a few are considered pathogenic in humans, namely *A. hydrophila, A. caviae*, and *A. veronii bv. sobria* (9). Symptoms of *Aeromonas-*associated gastrointestinal infection vary from diarrhea with loose stools to severe watery or bloody diarrhea with fever and abdominal pain lasting up to 4 weeks (10). The variability in *Aeromonas-*associated gastrointestinal symptoms has been correlated, at least partly, to the substantial variability in putative virulence-protein-encoding genes carried by different species and strains (9, 10). These organisms express an assortment of virulence factors, which allow them to colonize, invade, and infect numerous hosts. For instance, in addition to various biologically active surface structures and a repertoire of exoenzymes that digests cellular components, *Aeromonas* spp. produce several toxins targeting the intestinal mucosa (11). *Aeromonas* spp. secrete cytotoxic (the heat-labile Act) and cytotonic (the heat-labile Alt and the heat-stable Ast) enterotoxins and various hemolysins (including AerA and HlyA) (11). Indeed, genes encoding different toxins can be concomitantly found, in variable combinations, in the same strain (12, 13). The relationship between the occurrence of toxins genes and strains' pathogenicity in animals and humans is unclear since toxigenic strains have been identified in the environment and asymptomatic subjects (9, 10). Thus, the pathogenic potential of *Aeromonas* spp. seems multifactorial and complex and may result from products of different genes acting individually or collectively.

An additional point of concern is the growing incidence of resistance to diverse groups of antibiotics reported in Aeromonads. Several reports, mainly on *Aeromonas* spp. isolate from fish farms and the environment, have demonstrated a worrying incidence of drug-resistant strains correlated to the extensive use of antibiotics and other chemotherapeutics (14, 15). For instance, isolates of *Aeromonas* have shown relatively high resistance to β-lactam antibiotics, usually correlating with naturally occurring phenotypes of β-lactamases production (16). Furthermore, strains containing multiple antibiotic resistance (MAR) have been isolated from patients' stools and the environment. However, antimicrobial resistance seems to differ between strains isolated from different geographic environments but also compared to those from clinical sources.

In this study, we aimed to determine the incidence of *Aeromonas* spp. in human diarrheic stool specimens collected at the Microbiology Unit in North Eastern Italy. We characterized the isolated strains regarding their antimicrobial resistance, enterotoxin armamentarium, and pathogenicity mechanisms.

## RESULTS

### Prevalence of *Aeromonas* spp. in diarrheal stools

A total of 6,570 consecutive stool specimens collected from patients suffering from diarrhea were examined at the Microbiology Laboratory of Padua University Hospital in 2021. One hundred sixty-two fecal samples resulted positive for bacterial pathogens (Table 1), corresponding to 2.5% of the examined samples. *Aeromonas* spp. were identified as the causative agent of diarrhea in 40 patients, corresponding to 20.6% of subjects with a diagnosis of bacterial-mediated diarrhea, resulting as the second most common enteropathogen in our series (Table 2). As expected, *Campylobacter* sp. was the most common enteropathogen identified in patients with diarrhea, whereas *Salmonella enterica* and *Yersinia* spp. were less frequent (Table 2). The incidence of stool specimens positive for Aeromonas from patients suffering from diarrhea was comparable between 2021 and 2020, suggesting that the prevalence of this enteropathogen in our area is relatively stable (Tables 2 and 3).

**TABLE 1:** Overall picture of coproculture at Microbiology Laboratory of Padua University Hospital

	2020	2021
Fecal exams	6,421	6,570
Bacterial pathogen positive	158 (2.4%)	162 (2.5%)

**TABLE 2:** Enteropathogens isolated in diarrheal fecal samples

	2020	2021
*Aeromonas* spp.	39	40
*Campylobacter* spp.	82	87
*Salmonella* spp.	31	30
*Yersinia* spp.	6	5
Total	158	162

**TABLE 3:** Characteristics of patients with Aeromonas-positive coproculture

Gender	2020 (%)	2021 (%)
Male	17 (44)	21 (52)
Female	22 (56)	19 (48)

Age	2020	2021
0–15	10	12
16–30	2	1
31–45	2	2
46–60	15	7
61–75	3	10
>75	8	7

The gender ratio (male:female) was 1.1 (21/19) among patients presenting with diarrhea caused by *Aeromonas* spp. (Table 3). Aeromonas infections are known to occur in all age groups; however, we observed a peak incidence in children younger than 15 years and adults of 45 years and older (Table 3).

**Fig 1:**
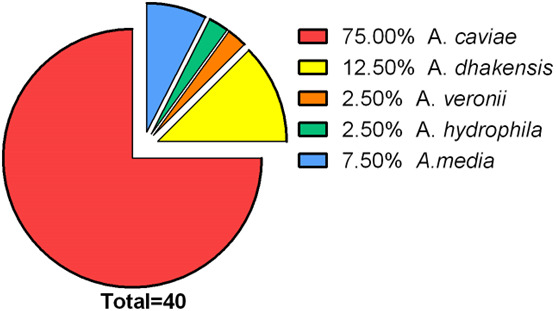
*Aeromonas* spp. in diarrheic patients. Prevalence of *Aeromonas* species isolated from identified with molecular methods in patients with diarrhea.

Finally, since the conventional biochemical method (VITEK2 Compact) appeared to provide unsatisfactory identification at the species level, we performed housekeeping gene sequencing with phylogenetic analysis (17, 18). Among the isolates recovered from feces of diarrheic patients in 2021, *A. caviae* was the most prevalent (75%), *A. dhakensis* represented about 12.5% of isolates, *A. media* 7.5%, whereas other species such as *A. veronii* and *A. hydrophyla* were rarely isolated (Fig. 1).

### Phylogenetic analysis of *Aeromonas* isolates

**Fig 2:**
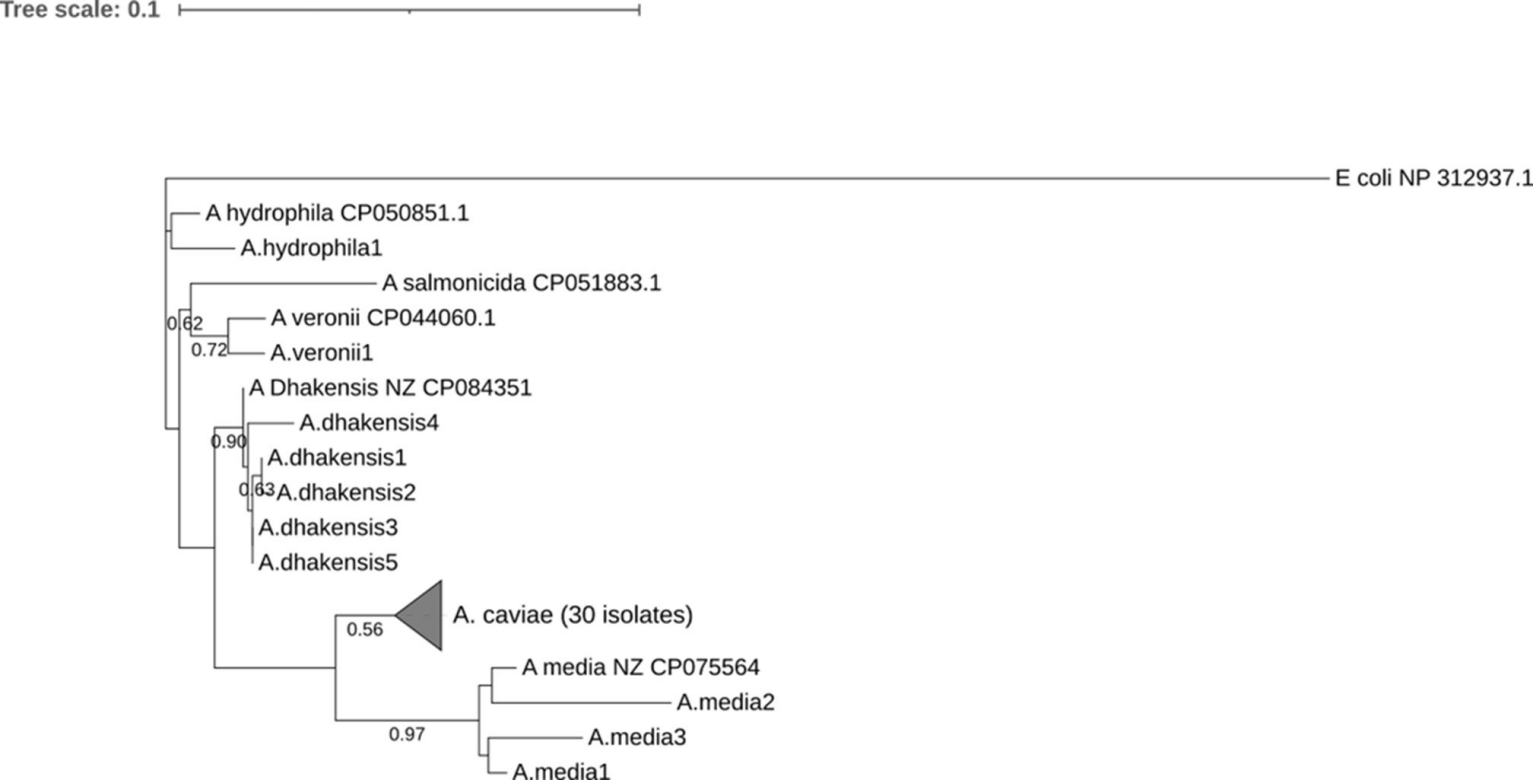
Phylogenetic analysis. The phylogenetic tree resulting from the alignment of sequence obtained from the PCR of the *rpoB* housekeeping gene. The reference sequences were extracted from GenBank and were included in the tree.

A multiple sequence alignment was built with the sequences obtained from the PCR of the *rpoB* housekeeping gene, followed by a phylogenetic analysis (Fig. 2). One reference sequence extracted from GenBank was included for each detected species (accession numbers OQ330882–OQ330921). The tree shows a good clustering among the *Aeromonas* spp., although with different levels of bootstrap consistency. *A. caviae*, the species most frequently detected, shows a relatively low clustering value (56%) (SI Fig. 1), which is affected by the high heterogeneity among sequences. The other *Aeromonas* spp. show a lower degree of intraspecies diversity, but the small sample sizes of these groups limit further considerations.

### Susceptibility to antimicrobials of *Aeromonas* clinical isolates

**Fig 3:**
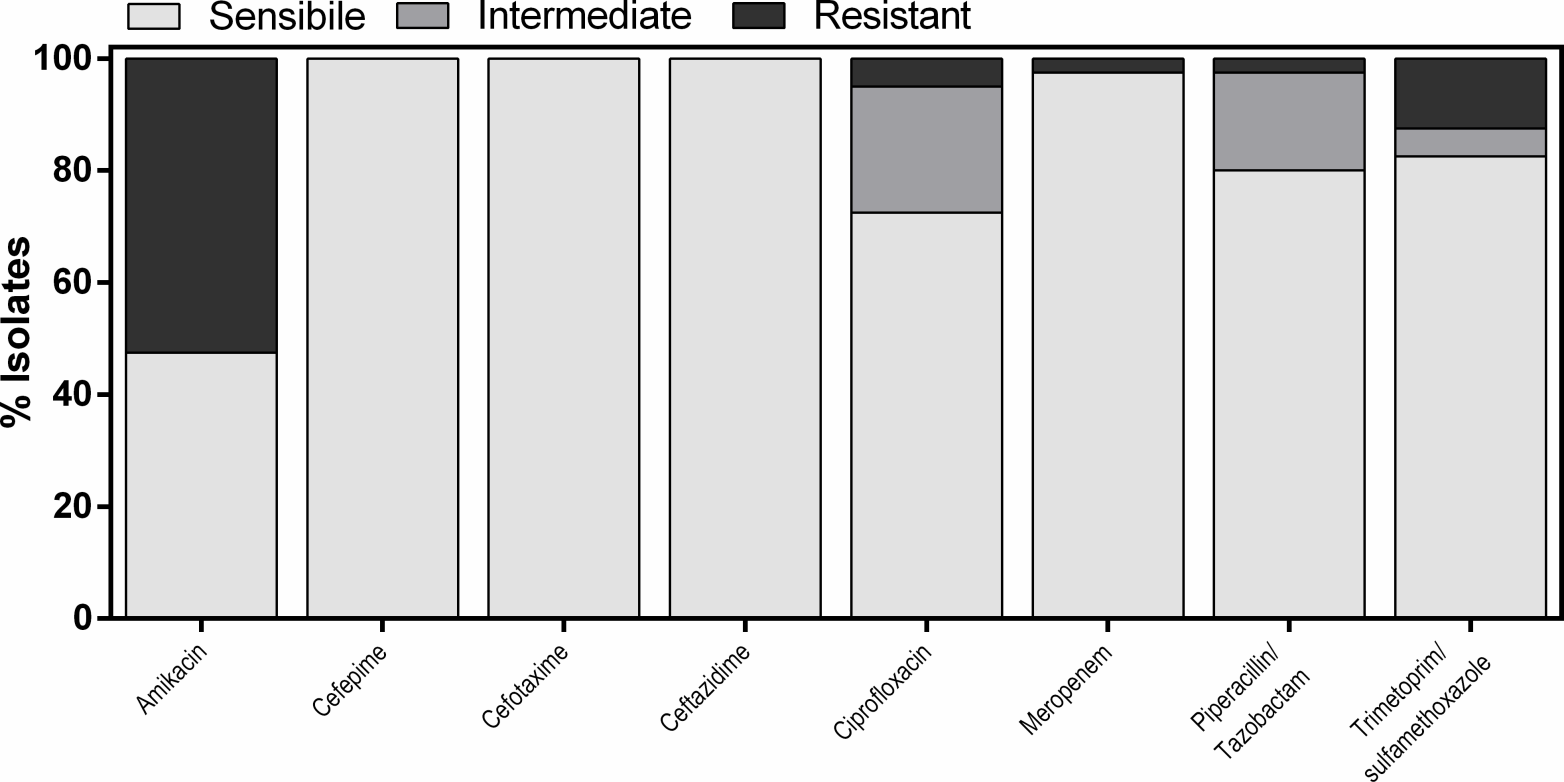
Susceptibility of *Aeromonas* isolates to antimicrobial agents. Antimicrobial susceptibility was assessed by Vitek2 assay. Results are reported in percentage. Intermediate corresponds to a microorganism categorized as “Susceptible, Increased exposure” following EUCAST guidelines.

Resistance profiles of the *Aeromonas* isolate against eight antimicrobial agents are shown in Fig. 3. All the bacterial isolates were susceptible to cefepime, cefotaxime, and ceftazidime; however, the susceptibility to other antimicrobial agents varied and 65% of *Aeromonas* strains showed resistance to one or more antibiotics. Resistance was most prevalent for amikacin, given that 52.5% of strains were unresponsive. Only 5% and 12.5% of *Aeromonas* were resistant to ciprofloxacin or trimethoprim-sulfamethoxazole, respectively. Resistance to meropenem, a carbapenem antibiotic, was barely detectable. MAR patterns were reported in 7.5% of the strains.

### Occurrence of virulence genes in *Aeromonas* clinical isolates

**Fig 4:**
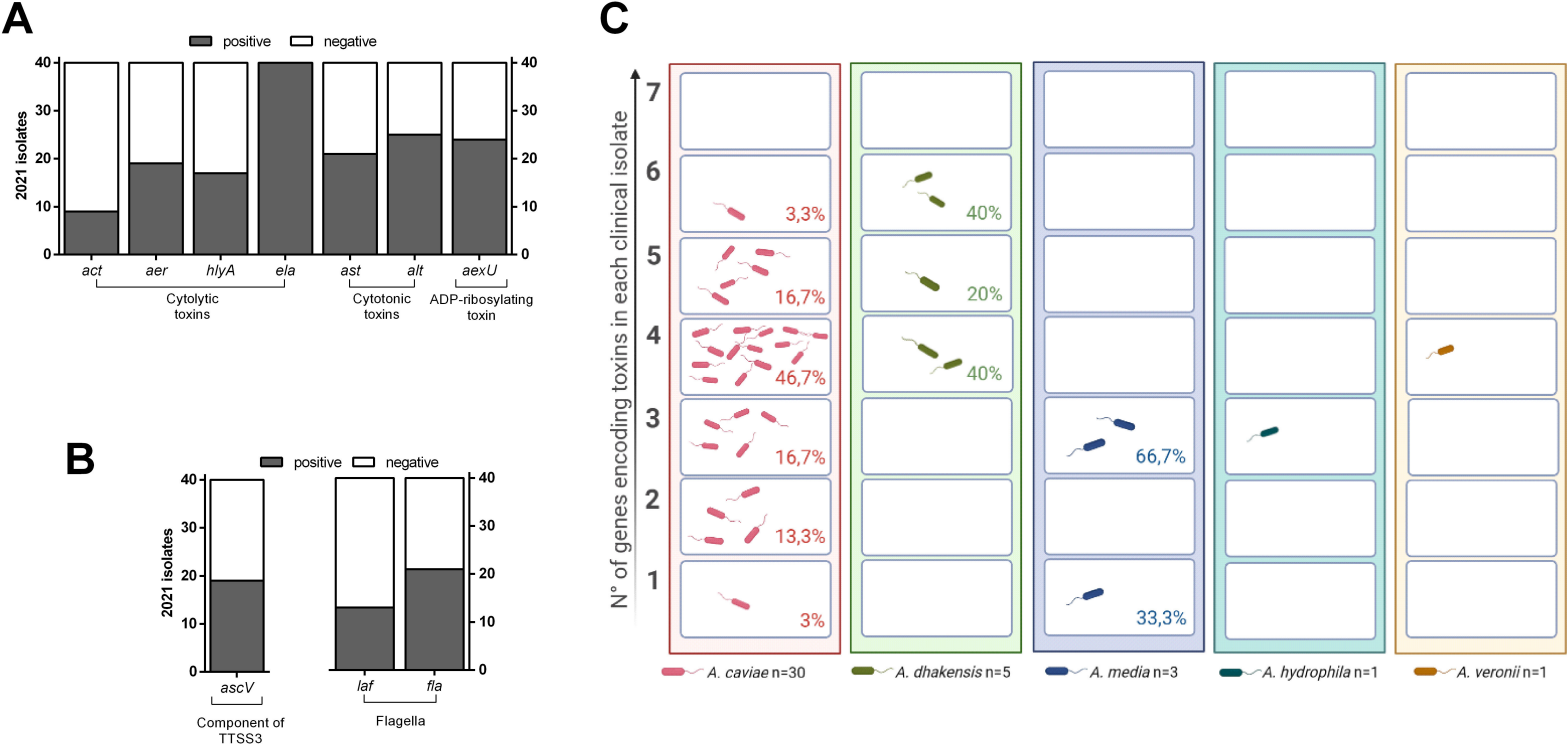
Distribution of virulence genes by species in *Aeromonas* strains isolated from patients with diarrhea. DNA purified from each strain was subjected to PCR for the specified genes; amplicons were visualized using Gel Doc EZ System. (**A**) Enumeration of presence or absence in each *Aeromonas* isolates of genes involved in the production of cytolytic toxins*—act, aer, hlyA, ela*, cytotonic toxins*—ast, alt*, ADP-ribosylating toxin*—aexU*. (**B**) Evaluation of presence/absence of genes coding for a component of type III secretion systems*—ascV*, lateral flagella*—laf*, and polar flagella*—fla*. (**C**) Illustration of numbers of toxins genes in each isolate and their representation of distributions among different species.

Forty *Aeromonas* isolates were screened for the presence of virulence genes. Molecular characterization involved assessment for elastase (*ela*), hemolysin A (*hlyA*), cytotoxin (*act*), aerolysin (*aerA*), ADP-ribosylating toxin (*aexU*), cytotonic heat-labile (*alt*), and cytotonic heat stable (*ast*) enterotoxin genes (Fig. 4A and B) (18). All *Aeromonas* strains isolated from diarrheic feces carried one or more toxin genes (Table 4). The virulence genotypes were found in different combinations: 26/40 (65%) possessed four or more virulence genes, whereas 8/40 isolates (20%) carried three virulence genes, and only 6/40 (15%) possessed two or one virulence gene (Table 4; Fig. 4). The most common enterotoxin genes in *Aeromonas* isolates were *ela* (100% isolates) and *alt* (65% isolates), whereas the least common (9/40) was the *act* gene (Table 4; Fig. 4). Toxin genes were not distributed evenly among *Aeromonas spp*. All *A. dhakensis* isolates possessed both *aer* and *hlyA* genes, as opposed to *A. caviae* (4/30, Tables 5 and 6). None of the clinical isolates possessed all the seven investigated genes (Fig. 4). Interestingly, we observed a different distribution between species regarding gene-coding toxins (Fig. 4). In *A. caviae*, we observed that almost 80% of isolated strains possessed four or fewer of the seven investigated toxin genes; only 6/30 *A caviae* held 5 or 6 of them (Fig. 4). *A. dhakensis* strains possessed four or more genes encoding for toxins (Fig. 4), appearing as the most virulent specie. Finally, *A. media* strains seem the least virulent since three or fewer genes for toxins were detected (Fig. 4).

**TABLE 4:** Virulence genes in *Aeromonas* clinical isolates

	*ast*	*alt*	*aer*	*act*	*hlyA*	*ela*	*aexU*	*ascV*	*laf*	*fla*
*A. caviae1*	+	+	−	−	+	+	−	−	−	+
*A. caviae2*	+	+	+	−	−	+	+	+	+	−
*A. caviae3*	−	−	−	−	−	+	−	+	−	+
*A. caviae4*	−	−	−	−	+	+	+	−	−	+
*A. caviae5*	+	+	+	−	−	+	−	−	−	−
*A. caviae6*	−	−	+	−	+	+	−	−	−	+
*A. caviae7*	−	−	+	−	−	+	+	+	−	+
*A. caviae8*	+	−	−	+	+	+	-	+	−	−
*A. caviae9*	+	+	−	−	−	+	−	+	−	+
*A. caviae10*	+	−	−	+	−	+	−	+	+	+
*A. caviae11*	+	+	+	−	−	+	+	−	−	+
*A. caviae12*	+	−	−	−	−	+	−	−	−	−
*A. caviae13*	−	−	+	−	+	+	+	+	−	+
*A. caviae14*	−	+	+	−	+	+	+	+	−	+
*A. caviae15*	−	+	−	−	+	+	+	+	−	−
*A. caviae16*	−	-	−	−	−	+	+	+	−	+
*A. caviae17*	−	+	−	−	+	+	−	+	−	+
*A. caviae18*	−	+	−	−	+	+	−	+	+	+
*A. caviae19*	+	+	−	−	−	+	+	+	−	−
*A. caviae20*	+	+	+	+	+	+	+	−	+	+
*A. caviae21*	+	+	+	+	−	+	+	−	+	+
*A. caviae22*	+	+	−	+	−	+	−	−	−	−
*A. caviae23*	+	+	−	−	+	+	+	−	−	+
*A. caviae24*	−	−	−	−	−	+	+	+	−	−
*A. caviae25*	−	−	+	−	−	+	+	+	+	−
*A. caviae26*	+	+	+	−	−	+	+	+	+	+
*A. caviae27*	−	+	−	−	−	+	+	+	−	+
*A. caviae28*	+	+	−	−	−	+	+	+	+	+
*A. caviae29*	+	+	+	−	−	+	+	−	−	−
*A. caviae30*	+	+	−	+	−	+	+	−	+	+
*A. hydrophila1*	−	−	+	+	−	+	+	−	+	−
*A. dhakensis1*	−	+	+	−	+	+	−	−	+	−
*A. dhakensis2*	+	+	+	+	+	+	−	−	−	−
*A. dhakensis3*	−	−	+	−	+	+	+	+	−	−
*A. dhakensis4*	+	+	+	+	+	+	−	−	−	+
*A. dhakensis5*	−	+	+	−	+	+	−	+	+	−
*A. veronii1*	−	−	+	−	+	+	+	+	−	−
*A. media1*	+	+	−	−	−	+	−	−	−	−
*A. media2*	−	−	−	−	−	+	+	−	+	−
*A. media3*	+	+	−	−	−	+	+	−	−	−

**TABLE 5:** Distribution of virulence genes among *Aeromonas* spp

	*ast*	*alt*	*aer*	*act*	*hlyA*	*ela*	*aexU*	*ascV*	*laf*	*fla*
*A. caviae*	17/30	19/30	12/30	6/30	11/30	30/30	19/30	16/30	9/30	17/30
*A. hydrophila*	0/1	0/1	1/1	1/1	0/1	1/1	1/1	0/1	1/1	0/1
*A. dhakensis*	2/5	4/5	5/5	2/5	5/5	5/5	1/5	2/5	2/5	1/5
*A. veronii*	0/1	0/1	1/1	0/1	1/1	1/1	1/1	1/1	0/1	0/1
*A. media*	2/3	2/3	0/3	0/3	0/3	3/3	2/3	0/3	1/3	0/3
Total	21/40	25/40	19/40	9/40	17/40	40/40	24/40	19/40	13/40	18/40

To evaluate the presence of the type III secretion system (T3SS) such as AexU and AexT (13), which injects toxins into target cells, we investigated the presence of the *ascV* gene that encodes for an inner membrane component of the T3SS apparatus. *AscV* gene was detected in 47.5% (19/40) of *Aeromonas* clinical isolates (Fig. 3B).

Since the flagella in *Aeromonas* spp. are considered necessary to adhere to biotic or abiotic substrates to form a biofilm, we investigated the presence of the *laf* and *fla* genes encoding lateral and polar flagella, respectively (19, 20). As reported in Table 5, 13/40 strains carried *laf* gene, whereas 21/40 possessed the *fla* gene (Fig. 3B).

### Biofilm formation by *Aeromonas* clinical isolates

**Fig 5:**
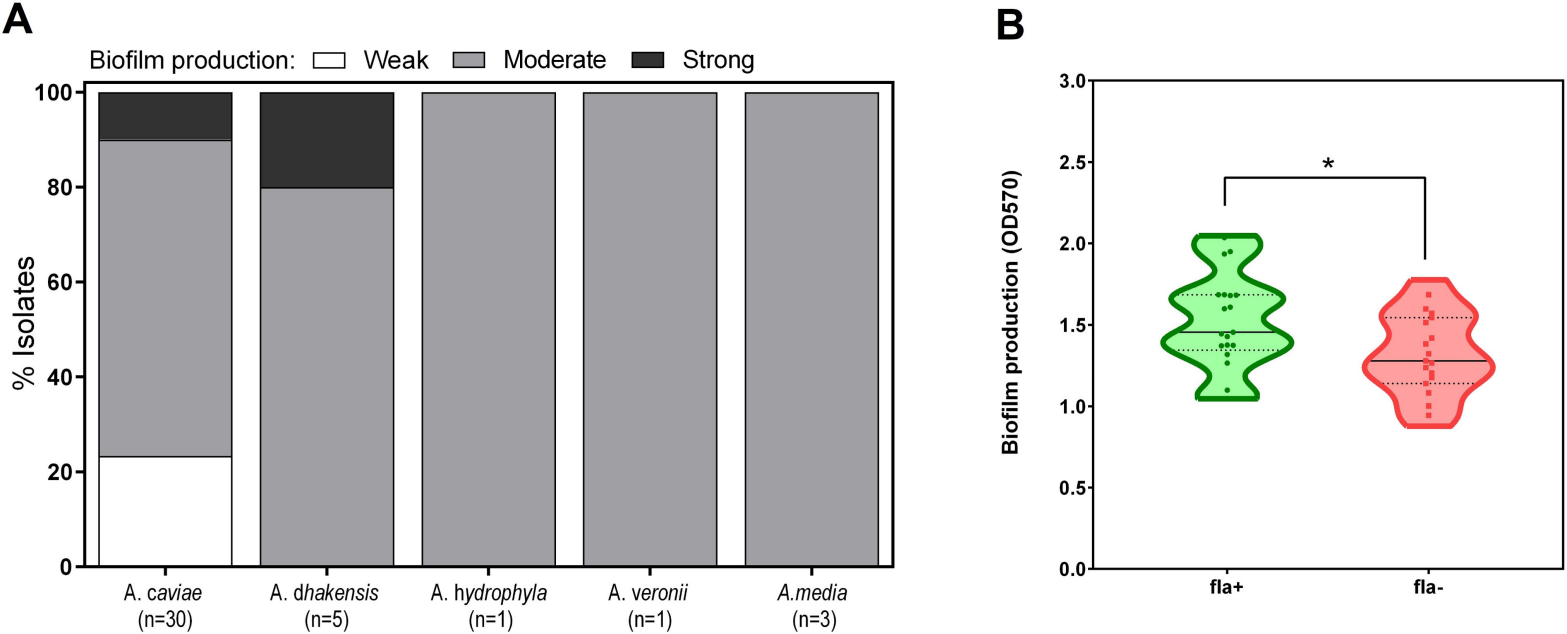
Biofilm formation of *Aeromonas* strains isolated from patients with diarrhea. *Aeromonas* cultures were grown into a 96-well plate at 37°C for 48 h. After incubation, biofilms were stained with crystal violet and biofilm biomass was measured by OD at 570 nm. (**A**) The strains were classified as weak, moderate, or strong biofilm producers. (**B**) The *Aeromonas* isolates produce a different biofilm in the presence or absence of *fla* gene. *P value < 0.05.

*Aeromonas* spp. can adhere to biotic or abiotic surfaces and form biofilms, a key virulence factor (21). Therefore, we determined the ability of *Aeromonas* clinical isolates to produce biofilm in static conditions. Although all strains could form biofilms, they had distinct productivities (Fig. 5A). The strains were classified as weak, moderate, or strong biofilm producers (Fig. 5A). Of the *Aeromonas* strains isolated from diarrheic feces, only 4 (10%) were strong biofilm producers, whereas 29/40 (72.5%) resulted in moderate biofilm producers (Table 6). Interestingly, the presence of *fla* gene improved biofilm formation (Fig. 5B).

**TABLE 6:** Biofilm production by different *Aeromonas* isolates

	Not forming	Weak	Moderate	Strong
*A. caviae*	-	7/30	20/30	3/30
*A. hydrophila*	-	0/1	1/1	0/1
*A. dhakensis*	-	0/5	4/5	1/5
*A. veronii*	-	0/1	1/1	0/1
*A. media*	-	0/3	3/3	0/3
Total	-	7/40	29/40	4/40

### Attachment and internalization of *Aeromonas* clinical isolates to Caco-2 cells

**Fig 6:**
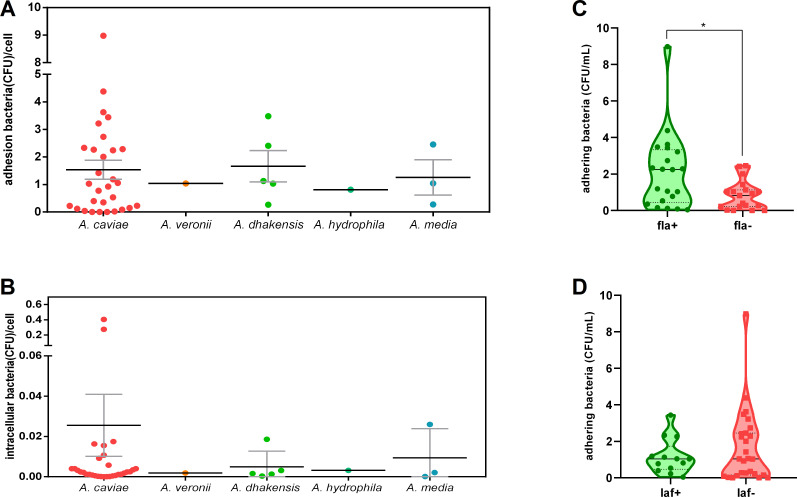
Adherence and invasion on Caco-2 cells of *Aeromonas* strains isolated from patients with diarrhea. Caco-2 monolayers were incubated with *Aeromonas* isolates (MOI 1:20) for 2 h at 37°C. Bacteria non-adhering to Caco-2 were removed by extensive washing, and (**A**) cells were lysed; aliquots were seeded on chocolate agar plates; adhering microbes were quantified by quantitative bacterial vital count assay. (**B**) Cells cultured for additional 2 h in complete medium containing gentamycin were lysed and invading microbes were quantified by quantitative bacterial vital count assay. Adhering or invading microbes were expressed as CFU/cell. All tests were performed in triplicate. (**C and D**) The influence of *fla* (**C**) or *laf* (**D**) genes on adhesion ability of *Aeromonas* isolates. *P value < 0.05.

To evaluate how *Aeromonas* strains interact with human intestinal epithelial cells, we first evaluated attachment and internalization to Caco-2 monolayers. Among the tested *Aeromonas* strains, only one presented an excellent attachment ability since almost 10 CFU/cell adhered to Caco-2 cells; 18/40 strains presented an adhesion ability lower than 1 CFU/cell (Fig. 6A). *A. caviae* strains exhibit an average adhesion of 1.5 CFU/cell. Nine isolates showed a high adhesion efficiency (>2 CFU/cell), whereas 13 strains showed low adhesion abilities (<1.0 CFU/cell) (Fig. 6A). Other *Aeromonas* spp. exhibited a similar ability to adhere to Caco-2 (Fig. 6A). Interestingly, we observed that the adhesion ability to human intestinal epithelial cells increases with the presence of *fla* gene that encodes for polar flagella (Fig. 6C); the presence or absence of gene coding lateral flagella (*laf*) does not influence the ability of clinical isolates to adhere to Caco-2 cells (Fig. 6D).

To assess the internalization ability of *Aeromonas* spp. into intestinal epithelial cells, we incubated Caco-2 cells exposed for 2 h to the bacteria with gentamicin to kill bacteria attached to the cells. The results summarized in Fig. 6B indicate that *Aeromonas* strains barely invade epithelial cells, suggesting that the adhesion to intestinal epithelium is the dominant phenomenon for this pathogen.

### *Aeromonas* clinical isolates stimulate inflammatory cytokines release from Caco-2

**Fig 7:**
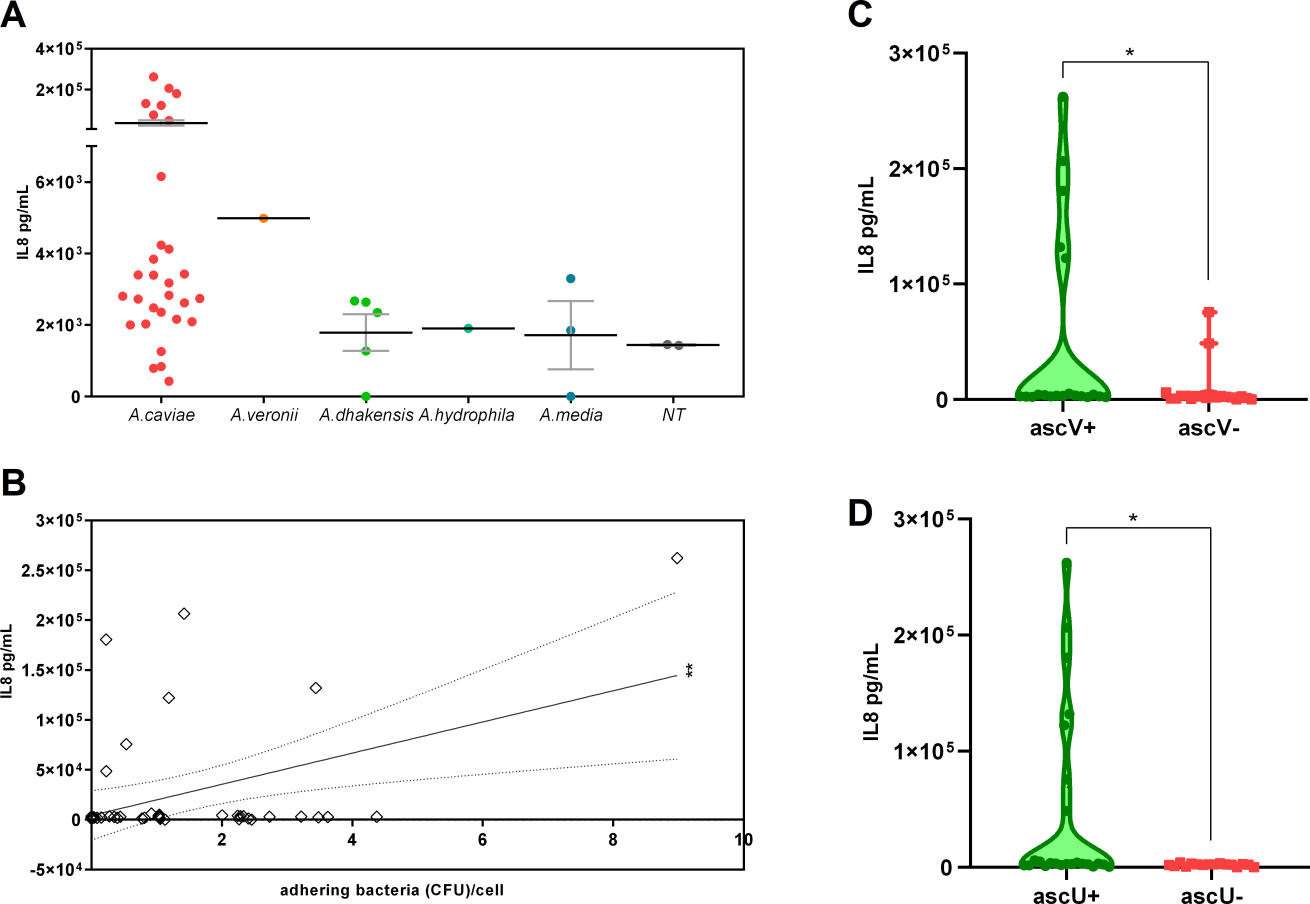
IL-8 in Caco-2 cells cultured with or without *Aeromonas* strains isolated from patients with diarrhea. (**A**) Caco-2 monolayers were incubated with *Aeromonas* isolates (MOI 1:20). After 2 h at 37°C culture medium was removed, the cells were washed and cultured in complete medium. Culture media were collected after 24 h. Levels of interleukin-8 (IL-8) were measured using commercially available enzyme-linked immunosorbent assay (ELISA) kits and expressed as pg/mL. All tests were performed in triplicate. (**B**) Correlation analysis between adhesion to Caco-2 cells and IL-8 release for each *Aeromonas* isolate *R* ^2^ = 0.1804; **P < 0.01. (**C and D**) Effect of *ascV* (**C**) or *aexU* (**D**) genes presence (*ascV/U+*) or absence (*ascV/U*−) on IL-8 induced production in Caco-2. *P < 0.05.

To determine whether the interaction of *Aeromonas* with intestinal epithelial cells triggers an inflammatory phenotype, we quantified IL-8 released by Caco-2 cells in response to *Aeromonas* strains isolated from patients with diarrhea. Among *A. caviae*, we again observed a dichotomic aptitude since 7 strains strongly induced IL-8 secretion, but 19 strains did not induce a pronounced cytokine release, in line with other *Aeromonas* spp. (Fig. 7A).

*Aeromonas* spp.-induced IL-8 release directly correlated with adhesion ability, suggesting that the direct interaction with intestinal epithelial cells is required to trigger inflammatory cytokines release (Fig. 7B). Moreover, the presence of the genes *ascV* and *aexU* stimulates Caco-2 cells in producing IL-8 (Fig. 7C and D).

### *Aeromonas* clinical isolates exert cytotoxic effects on Vero cells *in vitro*

**Fig 8:**
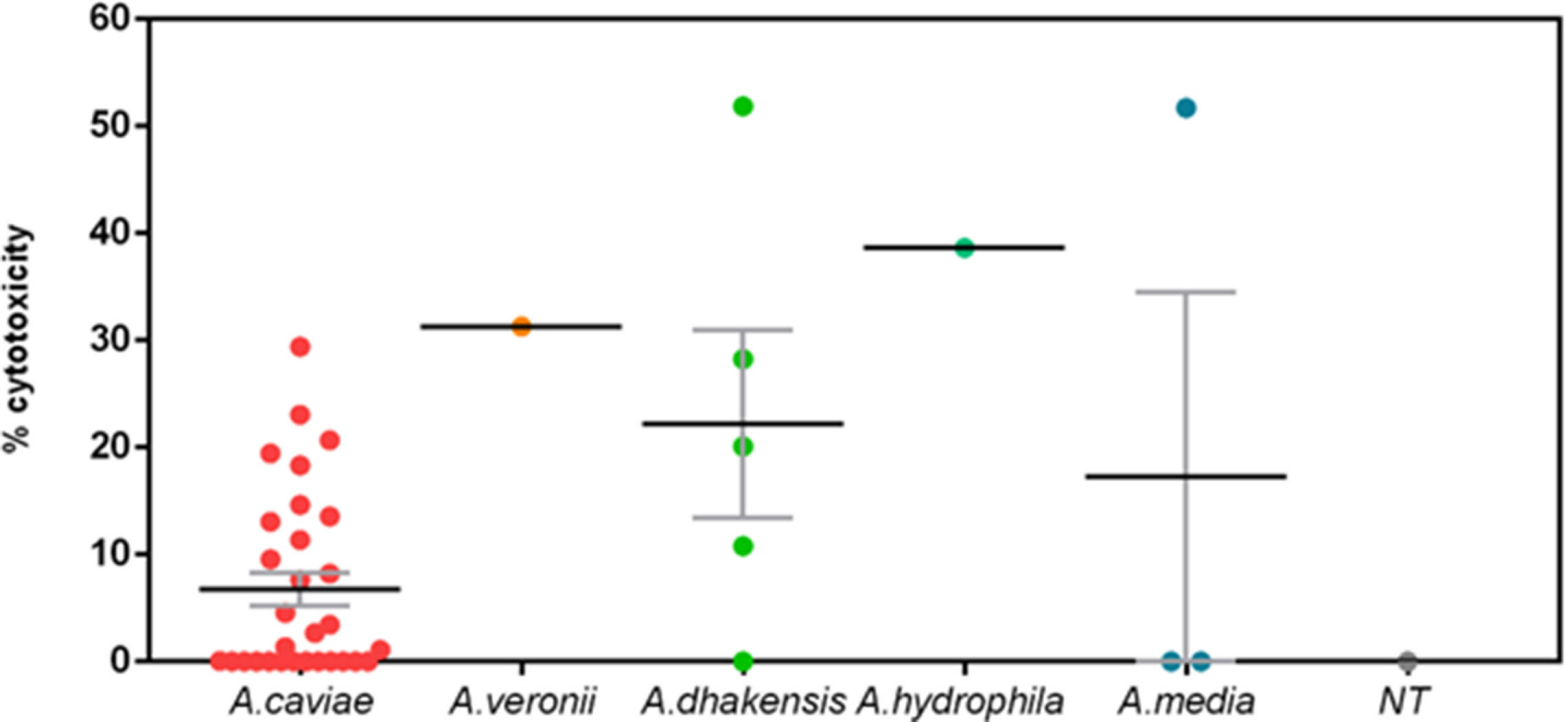
Cytotoxic effects of *Aeromonas* strains isolated from patients with diarrhea. Vero cells monolayers were incubated for 16 h with cell-free supernatant in a ratio of 1:10 vol/vol. Cell cytotoxicity was determined by crystal violet staining and measuring absorbance at 590 nm. The 0% of cytotoxicity was arbitrarily assigned to not-treated wells. All tests were performed in triplicate.

To measure the ability of *Aeromonas* strains to damage the epithelial cells directly, we determined the release of cytotoxic products. To this goal, we added *Aeromonas* culture-conditioned media to Vero cell monolayers. *A. caviae* strains reported a low cytotoxic effect since 21/30 isolates showed less than 10% cytotoxicity, whereas 9/30 exhibited a moderate cytotoxic effect ranging from 10 to 30% (Fig. 8). The other *Aeromonas* spp. showed an average cytotoxicity activity higher than the *A. caviae* group, even if the cytotoxic activity of the isolates was highly variable (Fig. 8).

### *AerA+ Aeromonas* strains damage the epithelial barrier

**Fig 9:**
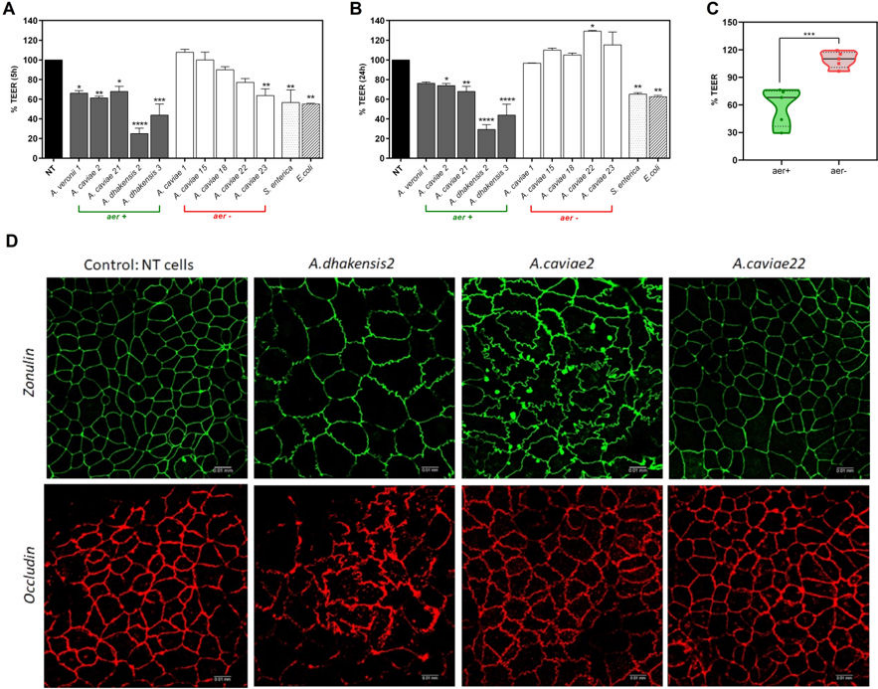
Effect of *Aeromonas* strains isolated from patients with diarrhea on transepithelial electrical resistance (TEER) in Caco-2 cell monolayers. (**A and B**) Post-confluent Caco-2 monolayers grown on transwell inserts were exposed to *Aeromonas* strains, either *Aer* + or *Aer*− (MOI 1:20). After 2 h at 37°C culture medium was removed, the cells were washed and cultured in complete medium containing antibiotics. TEER values were measured after 5 h (**A**) and 24 h (**B**). TEER was expressed as a percentage of resistance normalized to untreated monolayers (100%). *P < 0.05; **P < 0.01; ***P < 0.001; ****P < 0.0001 versus untreated Caco-2. (**C**) Effect of the presence/absence of *Aer* gene for each testing Aeromonas isolates on TEER of Caco-2 monolayers. ***P < 0.001. (**D**) Immunofluorescence localization of tight junction proteins zonulin-1 (ZO-1) and occludin (OC). Caco-2 monolayers were treated as described above and stained with anti-ZO and anti-occludin 5 h later. Samples were visualized using an inverted confocal microscope.

To assess the impact of *Aeromonas* strains on the integrity of the intestinal epithelial barrier, we measured transepithelial electrical resistance (TEER) in polarized Caco-2 monolayers grown in transwell apparatus (22). Polarized Caco-2 cells were apically exposed to different clinical isolates, either *aerA*+ or *aerA*−, or to enteropathogens known to disrupt epithelial barrier integrity, such as enteropathogenic *Escherichia coli* or *S. enterica*. In these experiments, we tested only *aerA− A. caviae* since all *A. dhakensis*, *A. hydrophila*, and *A. veronii* resulted *aerA+* (Table 4). All *Aeromonas aerA*+ caused a significant decrease in TEER 5 h post-exposure that persisted up to 24 h (Fig. 9), comparable to enteroinvasive *E. coli* and *S. enterica*. Indeed, *A. dhakensis* induced a more pronounced decrease in TEER than *A. caviae aerA*+ (Fig. 6). On the contrary, monolayers apically exposed to *Aeromonas aerA*− showed a modest transient decrease in TEER following 5 h incubation and exhibited resistances comparable to controls following 24 h (Fig. 9A and B). Therefore, *Aeromonas* spp.-induced damage of epithelial monolayer strongly increases with the presence of *aerA* gene, suggesting that the toxin aerolysin A damages the inter-epithelial cell junctions adhesion apparatus (Fig. 9C).

To investigate whether *Aeromonas*-induced intestinal barrier disruption is associated with changes in TJ protein expression or subcellular distribution, we performed an immunofluorescence assay evaluating ZO-1 and occludin in Caco-2 monolayers exposed to *aerA*− or *aerA*+ strains. Following exposure to *Aer*+ strains, we observed substantial disorganization in ZO-1 and occludin that appeared redistributed in microdomains in Caco-2 cells (Fig. 9D). On the contrary, we did not observe any change in ZO-1 and occludin immunoreactivity in Caco-2 monolayers exposed to *A.caviae22,* an *Aer*− strain (Fig. 9D).

## DISCUSSION

In diagnostic procedures, many coprocultures performed on diarrheic stool samples fail to identify a specific pathogen causing diarrhea (23). Even more sensitive techniques, such as antigen or nucleic acid detections in addition to coproculture, do not significantly improve the failure rate to discover a specific pathogen (23). The negative results can have various explanations, such as not infectious diarrhea and the inadequate handling and storage of fecal samples. However, the causative agent is usually not investigated as in the cases of viral infection or not yet identified enteropathogens. Therefore, in the United States, most diarrhea cases remain with unknown etiology (24, 25). Actually, several new enteric pathogens have been proposed in the past few years and *Aeromonas* spp. is among the newly identified human pathogens since this bacterial genus has been widely accepted as the etiological agent of infectious diarrhea (26, 27).

We undertook a prospective study at the Microbiology Unit of Padua University Hospital in Northeast Italy to define the frequency of identification of *Aeromonas* spp. in diarrheal disorders in our region and characterize the phenotype of the isolates. The factual incidence of *Aeromonas* in human gastrointestinal infections worldwide is unknown. However, it is well-accepted that it varies by geographical location and is higher in regions with low hygiene standards (10, 26, 27). In several studies worldwide, *Aeromonas* spp. have been isolated at a rate of 0.6–7.2% in patients with diarrhea, predominantly in infants and children (28, 29). In European countries, North America, and Israel, the incidence is estimated at 2% in patients with traveler’s diarrhea (28, 30, 31).

In contrast, *Aeromonas* spp. infection is the third cause of bacterial gastroenteritis in Spanish children, accounting for 2.5–4% of diagnoses (32 – 34). Similarly, in the USA, the incidence has been reported in 2.5% of children with diarrhea (34). In this study, 0.61% of diarrheic stools of patients were positive for *Aeromonas* spp., in agreement with previous reports and with the well-known notion that in industrialized countries, *Campylobacter* spp. (1.32% in our series) are the prevalent bacterial pathogens (35). Interestingly, in our series *Aeromonas* spp. were the second most common enteropathogen isolated from feces, being more numerous than *S. enterica* (0.46%). Therefore, *Aeromonas* spp. need to be considered as relevant human enteropathogens, like *Campylobacter* and *Salmonella* spp. The detection based on classical coproculture methods without selective medium could underestimate the actual incidence of this pathogen, even in “industrialized” countries (36). In our study, the Aeromonas spp. infection was monomicrobial in most cases (93%). In contrast, in previous studies evaluating traveler’s or children’s diarrhea, multiple pathogens were detected in a higher percentage of cases (31, 32, 37), probably due to differences in the study population. Our survey is free of the bias of patient recruitment and reveals a bimodal case distribution, with a peak in children younger than 15 years and a second large peak in subjects older than 45. According to previous reports, our study reported that 30% of the patients were infants and children (31).

In the current study, *Aeromonas* spp. were identified using the *rpoB* gene sequencing method, a procedure considered much more accurate than biochemical identification methods or 16S rRNA gene sequencing (17, 37, 38). In agreement with recent studies, *A. caviae* was recognized as humans' predominating species associated with diarrhea (32, 39). In contrast with previous reports, reporting *A. veronii* or *A. hydrophyla* as the second largest detected group (9, 31, 39); in our study, *A. dhakensis* represented the second most frequent group of *Aeromonas* strains isolated from diarrheic stool specimens. Overall, our data support the need for molecular tests to identify *Aeromonas* species to perform accurate epidemiological studies. The low clustering value observed among *A. caviae* isolates suggests different sources of infection (i.e., contaminated food) for each patient and seems to exclude clusters of infection or direct inter-human transmission.

*Aeromonas* spp. are intrinsically susceptible to most antibiotics active against non-fastidious Gram-negative bacilli, except for many beta-lactams due to chromosomally encoded β-lactamases (31). In line with previous studies, all strains were susceptible to third-generation cephalosporin antibiotics (cefotaxime and ceftazidime), and only two strains (5%) were resistant to the second-generation fluoroquinolone antibiotic, ciprofloxacin (31, 40, 41). Surprisingly, the majority of strains (52.5%) were resistant to aminoglycoside antibiotics (i.e., amikacin), in contrast to the high susceptibility previously reported for human isolates in Israel, China, and Malaysia (32, 42, 43). Since aminoglycoside-resistant strains have been reported mainly from aquatic sources, our data suggest that patients in our area might be exposed principally to *Aeromonas* strains originating from fish of aquaculture (42, 44). Recently, several reports described carbapenemase genes in this group of bacteria (45, 46). Carbapenemase-producing *A. hydrophila* has been identified by routine perirectal surveillance culture and in clinical isolates from various sources, including stools and polluted water (42, 43). However, we have not identified carbapenemase-producing *Aeromonas*, suggesting that these pathogens are not contributing to the spread of these genes in our area.

The mechanism of *Aeromonas* pathogenesis in the gut is complex and no single putative virulence-associated factor can be unequivocally pinpointed as responsible for GI diseases (44). Most studies characterizing virulence factors associated with *Aeromonas* pathogenicity have been performed in strains isolated from environmental sources such as polluted and drinking water (44). However, regardless of their origin, *Aeromonas* strains possess an impressive variety of virulence factors (44). In addition to structural components of the bacterial cell (i.e., LPS, pili, and flagella), *Aeromonas* strains can produce a variety of extracellular enzymes and toxins; as a result, the toxigenic profile is extremely mutable (1, 47, 48). In agreement with previous studies, also in the human isolates retrieved in North Eastern Italy, we observed high heterogeneity in the distribution of toxin genes among the isolates (31, 44, 49). Thus, our clinical isolates harboured at least one of the putative virulence genes *ast*, *alt*, *aer*, *act*, *hlyA*, *ela*, and *aexU*. The percentages of positivity for virulence genes obtained in our study were in line with food and environmental strains, as well as clinical isolates previously described (31, 44, 47). Interestingly, we observed a strong presence of virulence genes in *A. dhakensis* according to a previous study which pointed out the importance of this species due to a large number of virulence genes but also to its high rates of drug resistance and ability to cause both intestinal and extra-intestinal infections (39).

*Aeromonas* is characterized by the ability to produce biofilms on the biotic or abiotic surfaces that plays a crucial role in the colonization process and amplify antibiotic resistance. In agreement with previous reports, the present study demonstrates that clinical isolates had different abilities to form biofilms, even under the same experimental conditions (19). Moreover, biofilm formation depends on the specific strain and is not a general characteristic of a bacterial species or serotype (50). Moreover, by comparing biofilm biomass by diverse *Aeromonas* spp. for 48 h, our data support the view that functional polar flagella seem required for optimal adherence and biofilm formation, in line with most of the published studies (21, 51).

To study the interaction of *Aeromonas* clinical isolates with gut epithelial cells, we used the Caco-2 cells model, widely used to investigate pathogens' effects on GI mucosa, including *Aeromonas* spp. (9, 52, 53). Adhesion and invasion of bacteria to mucosal surfaces are a critical step in the pathogenesis of most GI infections. The adherence capacity of *Aeromonas* spp. to several human cell lines has been previously reported (54, 55). Compared to previous studies reporting adherence between 3.2 and 6.5 bacteria per epithelial cell of strains isolated from the polluted waterways, the Aeromonas isolates described in this study and obtained from diarrheic feces showed a slightly less adhering ability (8, 19, 52). In contrast with a previous study, we observed that the expression of the gene encoding for polar flagellum (*fla*), rather than for the gene encoding for lateral flagella (*laf*), correlates with the adhesion and colonization ability (19). Our clinical isolates, in agreement with previous reports on strains isolated from the environment and food, showed a low index of invasion (56, 57). The limited invasive ability of *Aeromonas* strains observed in this and previous studies suggests that the pathogenetic mechanism of *Aeromonas* spp. differs from that of “classical” invasive enteropathogens such as *E. coli*, *Shigella*, or *Salmonella* spp (53).

Although tissue-dependent responses have been reported, it is well accepted that *Aeromonas* spp. enhance several proinflammatory cytokine overexpression in animal models (9). In line with the reported ability of *Aeromonas* spp. to promote the activation of transcription factors and secretion of proinflammatory cytokines, our clinical isolates stimulate IL-8 release from Caco-2 cells (9). Intriguingly, *Aeromonas*-induced cytokine release required direct epithelial cell-pathogen contact, directly correlating to the extent of adhesion to epithelial cells.

Since our results point out that *Aeromonas* spp. inducing IL-8 release require a type III secretion system or the association with toxin AexU, they support the view that *Aeromonas* strains modulate inflammatory responses in intestinal epithelial cells by directly inoculating specific toxins (31, 58). Moreover, the amplitude of cytokine release was strain-dependent suggesting that the variable clinical presentation of *Aeromonas* GI infection might be determined by to different extent of proinflammatory cytokines secreted by the epithelial cells that amplify mucosal damage, such as in other infective diseases (53, 59, 60). However, further studies are required to correlate the severity of the clinical picture and the virulent armamentarium of *Aeromonas* spp. and to dissect the innate immune mechanisms engaged by the gastrointestinal system to detect and clear invading Aeromonas.

Impairment of TJs function, caused by gastrointestinal pathogens, is implicated in the pathology of gastrointestinal infections (61). In agreement with previous studies, we demonstrated that the aerolysin is the main effector of *Aeromonas*-induced barrier impairment, as bacteria strains lacking this gene failed to affect TJs function and integrity (62). Direct exposure of Caco-2 monolayers to *Aer*+ strains induced a fast drop in TEER along with TJ proteins redistribution in raffles caused by differential claudin/ZO-1 interactions (63). Indeed, aerolysin induces TJ protein redistribution via Ca^2^+ signaling thus producing actomyosin contraction, which in turn causes retraction and redistribution of TJ proteins forming membrane ruffles (62). *A. dhakensis* strains caused a more prominent and persistent decrease in transepithelial resistance than *A. caviae Aer*+. Intriguingly, *A. dhakensis* strains carried, in addition to *Aer* gene, the *hlyA* gene coding a toxin that, in inflammatory or inflammation-prone conditions, potentiates the leaky gut phenomenon, showing barrier-breaking effects (64). Thus, we support the view that various toxins contribute to damage to the epithelial barrier, eventually increasing the severity of clinical pictures (65).

In conclusion, this work demonstrates the epidemiologic relevance of the *Aeromonas* genus as the cause of infective diarrhea in North East Italy, both in children and adult subjects, with the significative presence of highly pathogenic strains. Phylogenetic analysis and antibiotic resistance pattern suggest that the major source of *Aeromonas* strains causing diarrhea in North Eastern Italy might derive from aquaculture fish, posing the need for more strict surveillance to improve food safety standards. *Aeromonas* strains possess a heterogeneous armamentarium of pathogenicity factors that allows the microbe to affect a wide range of human intestinal epithelial cells processes that justify the possibility of inducing diarrhea through different mechanisms and cause diseases of variable severity, as observed for other gastrointestinal pathogens (66). However, it remains to be determined whether specific genotype(s) are associated with clinical pictures of different severity (i.e., bloody or watery diarrhea) to implement the diagnostic approaches (i.e., search of specific genes in isolates) and therapeutic approaches for this relevant enteric pathogen.

## MATERIALS AND METHODS

### *Aeromonas* prevalence in diarrheal fecal samples

The isolation and identification of *Aeromonas* spp. were performed using standard microbiological procedures in diarrheic stool specimens submitted to the Microbiology Laboratory of Padua University Hospital, which provides services to a vast population in the Padua metropolitan area (North Eastern Italy) (67). Samples were collected between January 2021 and December 2021. All specimens were examined routinely for conventional enteropathogens, namely *Shigella*, *Salmonella*, *Yersinia,* and *Campylobacter* spp., identified by established methods (67). For the isolation of *Aeromonas* spp., the fecal specimens were diluted (1:10), and a loop of material was streaked on selective Shigella Aeromonas agar. The agar plates were incubated for 48 h at 25°C. Colonies morphologically suspected as *Aeromonas* were identified at the genus level using an automatic bacteriologic analyzer (VITEK2 Compact, BioMerieuX, France). Table 3 summarizes the gender and age of the patients and the month in which each strain was isolated. Strains were stored in Luria-Bertani (LB, Becton Dickinson) broth and glycerol mixture (70:30) at −80°C until genotypic and phenotypic tests were performed. For subsequent tests, *Aeromonas* strains were grown in LB broth at 37°C.

*Aeromonas* species were determined by amplifying and sequencing the RNA polymerase β subunit gene (*rpoB*), a housekeeping gene, following a previously published method (38). To amplify the sequence, we used the following primers Fw-GCAGTGAAAGARTTCTTTGGTTC and Rv-GTTGCATGTTNGNACCCAT. The PCR products were sequenced by Eurofins Genomics (Germany). Newly obtained sequences were compared to those available in the GenBank database, using the standard nucleotide–nucleotide BLAST program (BLASTN; http://www.ncbi.nlm.nih.gov) to establish their closest relatives. The sequences were submitted to the GenBank database under accession numbers OQ330882–OQ330921. A phylogenetic tree was generated using the Maximum Likelihood method with MEGA 11 (https://doi.org/10.1093/molbev/msab120) based on alignments from CLUSTAL W (10.1093/nar/22.22.4673).

### DNA extraction and PCR amplification

Strains kept at −80°C were streaked on selective Shigella Aeromonas agar, and then an isolated colony was inoculated in 10 mL of LB and grown overnight at 37°C with agitation (150 rpm/min). The culture was centrifuged (3,800 rpm for 10 min), and bacteria were incubated in 500 µL of Lysis Buffer (NaCl 10 mM, MgCl_2_ 3 mM, Tris–HCl 20 mM pH 7.4, 0.3% Nonidet P40, and 1.25% sucrose), 62.5 µL of SDS 10% and 20 µL of proteinase K 200 µg/mL for 1 h at 56°C with frequent vortexing. Total chromosomal DNA from *Aeromonas* spp. was purified using standard phenol/chloroform methods. The DNA was suspended in DNAse and RNAse-free water, quantified and subjected to PCR using primers and conditions described in Table 7. PCR products were electrophoresed in 2% wt/vol agarose gel and visualized using Gel Doc EZ System Bio-Rad.

**TABLE 7:** Primers and amplification conditions for PCR

Gene	Primer 5' → 3'	T melting (°C)	Length (bases)	References
*alt*	Fw**-** GCACGGCGTGACTTCGGTGA Rv-ACCGCGGTCTTGCAGTTGGG	60	576	This study
*aer*	Fw-GCCTGAGCGAGAAGGT Rv-CAGTCCCACCCACTTC	54	419	This study
*ast*	Fw-CGCCATCAACAGCTCGCCCA Rv-CGGGCCTCGTTGAGGAAGCG	60	212	This study
*hlyA*	Fw-CCACGCAAATTCATCACG Rv-ATCCTTGTTCACCTCGAC	56	1,079	68
*ela*	Fw-ACACGGTCAAGGAGATCAAC Rv-CGCTGGTGTTGGCCAGCAGG	62	510	68
*act*	Fw-CCGGGCTCGGCGTCCAATAC Rv-CCAGTTCGGGCGGTTGTCCG	60	819	This study
*aexU*	Fw-TGGTGAACCGGCGCAAAGTG Rv-ATATGAGCCAGCGCAGCCAG	60	789	13
*ascV*	Fw-ATGGACGGCGCATGAAGTT Rv-TATTCGCCTTCACCCATCCC	60	710	68
*laf*	Fw-GGTCTGCGCATCCAACTC Rv-GCTCCAGACGGTTGATG	56	550	19

### Biofilm quantification

*Aeromonas* strains were grown overnight in LB at 37°C with agitation (150 rpm/min), and then cultures were diluted 1:100 in fresh LB medium and incubated for additional 2 h at 37°C. The cultures were centrifuged and suspended in Brain Heart Infusion broth (BHI) at 10^8^ CFU/mL. About 200 µL of bacterial suspensions was placed into 96-well polystyrene microtiter plates and incubated at 37°C in an aerobic environment under static conditions. The biofilm quantification was conducted utilizing the crystal violet method with some modifications (69). Following 48 h of incubation, the wells were emptied and washed three times with sterile phosphate-buffered saline (PBS) to remove planktonic cells. Adhering cells were fixed with ethanol, and the biofilm was incubated with crystal violet 0.1% wt/vol in the dark at 37°C. After 30 min, the excess of crystal violet was removed, and the plate was left to dry for 24 h at 37°C. Finally, 100 µL of acetic acid 30% vol/vol was added to solubilize the dye. Optical density (OD) at 570 nm was recorded as a measure of biofilm biomass using a microplate reader (Perkin Elmer Victor X2 Multilabel Microplate Reader).

### Eukaryotic cell culture

The human intestinal epithelial cells (Caco-2, ATCC HTB-37) and African green monkey kidney cells (Vero cells, ATCC CCL-81) were cultured in Dulbecco’s modified Eagle’s medium (DMEM) (Gibco) supplemented with 10% fetal calf serum (FCS, Sigma-Aldrich), 25 mM HEPES (Sigma-Aldrich), 100 U/mL penicillin (Sigma-Aldrich), and 100 µg/mL streptomycin (Sigma-Aldrich). The cells were grown at 37°C in a humidified atmosphere of 5% CO_2_, 95% air. The culture medium was replaced every 48–72 h, and the cultures were split when cells reached 75% confluence. For experiments, the adherent cells were washed two times with sterile Hanks Balanced Salt Solution (HBSS, Gibco), detached with trypsin solution (0.04%, Gibco), resuspended at 10^6^ /cells/mL, and then seeded into appropriate cell culture plates. Cells were used for experiments when they reached 95–100% confluence.

### Adhesion and invasion of Caco-2 monolayers

The adhesion of *Aeromonas* strains to Caco-2 monolayers was assessed as previously described (52). Confluent Caco-2 monolayers in 24 wells/plate were washed two times with warm HBSS and then incubated with 500 µL of DMEM w/o antibiotics. *Aeromonas* strains cultured in LB-broth overnight at 37°C with agitation (150 rpm/min) were collected by centrifugation and adjusted to a concentration of 10^8^ CFU/L in DMEM w/o antibiotics. Then bacteria were added to Caco-2 monolayers (MOI 1:20) and incubated at 37°C in a humidified atmosphere containing 5% CO_2_, with slight shaking to promote cell/bacteria interaction. After 1 h, non-adhering *Aeromonas* were removed by extensive washes with warm HBSS. Epithelial cells were then detached, collected, and lysed to quantify the bacteria adhering to Caco-2 monolayers. Living bacteria were enumerated by quantitative bacterial vital count assay. Serial dilutions of lysed epithelial cells were seeded on LB agar plates and incubated at 37°C. After 16 h, the CFU of bacteria *per* cell was calculated. To quantify the bacteria invading Caco-2 monolayers, following extensive washes with HBSS, the cells were incubated in DMEM containing gentamycin. After 2 h, living bacteria associated with Caco-2 cells were enumerated by quantitative bacterial vital count assay as described above.

### Cytokine release

*Aeromonas* strains-induced cytokine release from Caco-2 monolayers was performed as previously described for enteropathogens (70). *Aeromonas* strains cultured in LB-broth overnight at 37°C with agitation (150 rpm/min) were centrifuged, adjusted to a concentration of 10^8^ CFU/mL and added (MOI 1:20) to confluent Caco-2 monolayers. Monolayers and bacteria were co-incubated at 37°C in a humidified atmosphere containing 5% CO_2_. After 2 h, the culture media was removed, monolayers were washed two times with warm HBSS, and then cells were incubated with DMEM containing gentamycin (50 µg/mL). Conditioned media were collected after additional 16 h and stored at −20°C until IL-8 was measured by a commercially available ELISA kit (eBioscienzeTM).

### Effect of *Aeromonas* clinical isolates on transepithelial electrical resistance

Caco-2 cells were seeded on Transwell polyester membrane cell culture inserts (transparent PET membrane: 1.0 cm^2^ growth surface area, 0.4 µm pore size; BD Falcon) in 24-well plates and incubated with DMEM medium w/o antibiotics. The culture medium was replaced every 48 h until the confluence. As previously described, the TEER values were measured daily in HBSS (22). Monolayers were used when TEER values remained stable for three consecutive days. Then, *Aeromonas* clinical isolates cultured overnight at 37°C in LB were placed (MOI 1:20) in the upper chamber of Transwell inserts and left in contact with monolayers. After 2 h, the culture medium was replaced with fresh DMEM containing gentamycin (50 µg/mL), and Transwells were incubated at 37°C. TEER values were measured after 5 and 24 h. TEER values of Caco-2 monolayers exposed to bacteria were expressed as a percentage of monolayer resistance not exposed to bacteria.

### Evaluation of *Aeromonas* spp. effects on tight junction proteins

Caco-2 cells were seeded on glass coverslips in six-well plates and kept in culture for 7–10 days after reaching confluence. Then, cells were washed and incubated with *Aeromonas* strains (MOI 1:20) in DMEM without antibiotics. The culture medium was removed after 2 h and substituted with fresh DMEM supplemented with FBS and gentamycin to eliminate residual *Aeromonas*. After 5 or 24 h, epithelial cells were fixed in 4% wt/vol paraformaldehyde (PFA) for 15 min. Cells were washed three times with PBS 1× and incubated with anti-zonula occludens (ZO)-1 or anti-occludin polyclonal antibody (Invitrogen) in PBS/0.2% Triton X100/2% BSA (PBS-T). After 1 h, cells were washed three times in PBS-T (10 min each), and monolayers were incubated with proper secondary antibody FITC labeled. After 30 min, following extensive washing, in PBS/0.2% Triton X-100 (3 × 10 min). Samples were visualized with a Nikon A1RSi laser scanning inverted confocal microscope equipped with NIS-elements advanced research software using 40× ocular objectives. Microscope settings were established to collect images below saturation and were kept constant for all image acquisition.

### Cytotoxic effect of *Aeromonas* clinical isolates on Vero cells

*Aeromonas* isolates (10^8^ CFU/mL) were grown overnight in LB-broth at 37°C with agitation (150 rpm/min). The culture was centrifuged and supernatant sterile filtered using a 0.22-µm syringe filter. Cell-free supernatant was added to confluent Vero monolayers at the ratio of 1:10 vol/vol. Cell cytotoxicity was determined by crystal violet staining following previously reported methods (71). After 16 h of incubation with the supernatants, cells were fixed in PFA 4% for at least 20 min. Cells were then washed with PBS 1× three times. Plates were stained with a 0.4% crystal violet solution in methanol for 30 min. After three additional washing, stained cells were solubilized with 30% acetic acid. Absorbance at 590 nm was measured using plate reader (Varioskan Lux Reader, Thermo Fisher Scientific). No treated wells were arbitrarily assigned 0% of cytotoxicity. All tests were performed in triplicate.

### Statistical analysis

The data were analyzed using one-way analysis of variance, followed by Bonferroni multiple comparisons using GraphPad Prism (version 8.0) in Fig. 9A–B. The correlation diagram and regression line were conducted using GraphPad by calculating Pearson *R* ^2^ in Fig. 7C. In Fig. 5B–6C/D–7B/D–9C, were used *t* test analysis to compare two different groups. *P* values of ≤0.05 were taken as statistically significant.

## Data Availability

The *rpoB* sequences generated and analyzed during the current study are available from the corresponding author on request or are available in the GenBank repository with accession numbers from OQ330882 to OQ330921.

## SUPPLEMENTAL MATERIAL

Fig. S1 spectrum.00807-23-s0001.pdf The complete phylogenetic tree resulting from the alignment of sequence obtained from the PCR of the *rpoB* housekeeping gene.

ASM does not own the copyrights to Supplemental Material that may be linked to, or accessed through, an article. The authors have granted ASM a non-exclusive, world-wide license to publish the Supplemental Material files. Please contact the corresponding author directly for reuse.
