# Supplementary material for: Prevalence and virulence potential of Aeromonas spp. isolated from human diarrheal samples in North East Italy
Source: Microbiol Spectr. 2023 Oct 19;11(6):e00807-23. doi: 10.1128/spectrum.00807-23 (PMC10715124; doi:10.1128/spectrum.00807-23)
Supplement: Fig. S1 — The complete phylogenetic tree resulting from the alignment of sequence obtained from the PCR of the rpoB housekeeping gene. [file spectrum.00807-23-s0001.pdf]

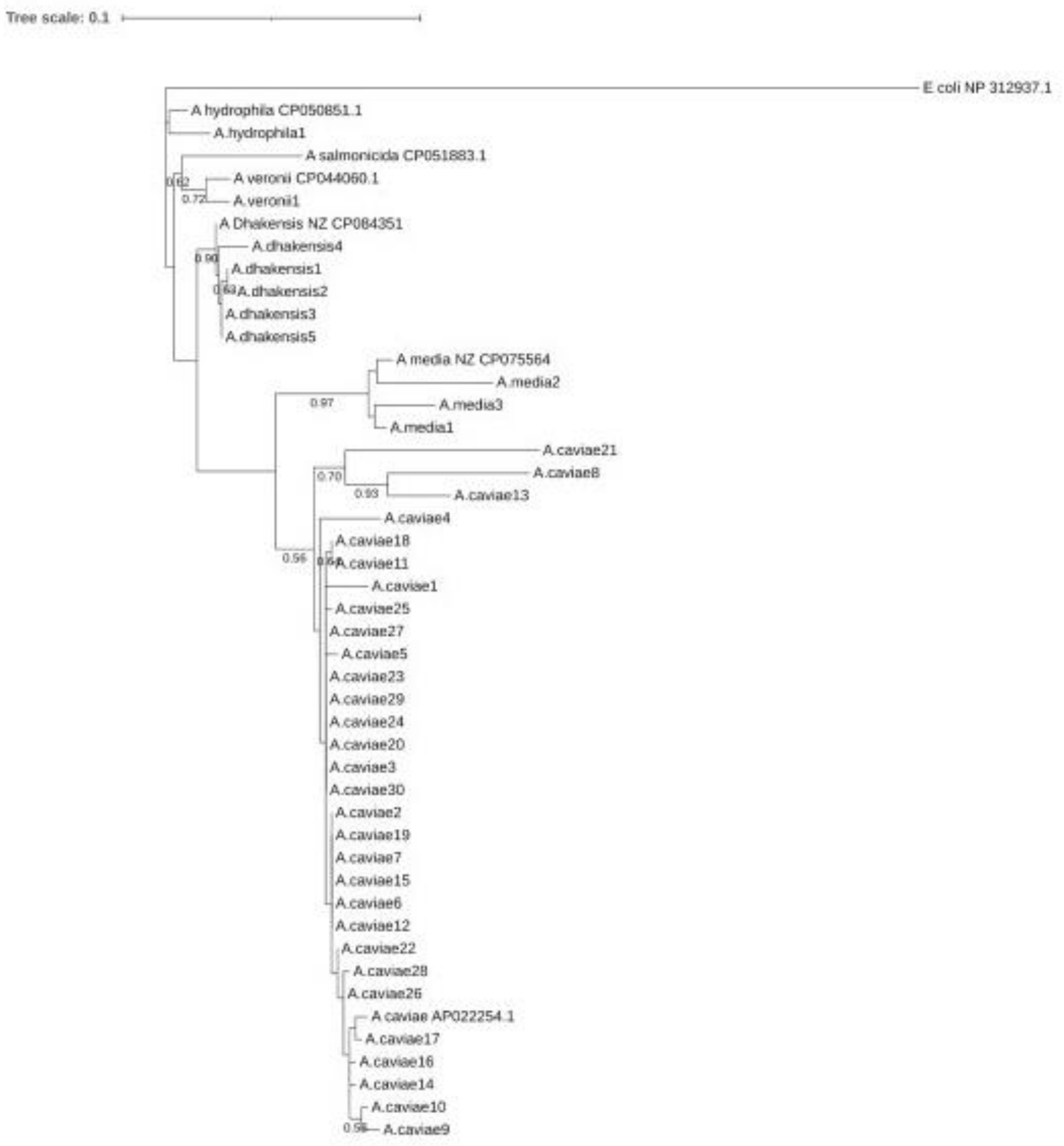

S1 Figure. Phylogenetic analysis. The phylogenetic tree resulting from the alignment of sequence obtained from the PCR of the rpoB housekeeping gene. The reference sequences were extracted from GenBank and were included in the tree
